# Prevalence of Obesity: A Public Health Problem Poorly Understood

**DOI:** 10.3934/publichealth.2014.2.109

**Published:** 2014-06-20

**Authors:** Theresa A. Nicklas, Carol E. O'Neil

**Affiliations:** 1 USA USDA/ARS Children's Nutrition Research Center at Baylor College of Medicine, Department of Pediatrics, 1100 Bates Avenue, Houston, Texas 77030, USA; 2 Louisiana State University Agricultural Center, 261 Knapp Hall, Baton Rouge, Louisiana 70803, USA

**Keywords:** obesity, Sugar-Sweetened beverages, soda tax, dietary recommendations, total diet

## Abstract

This review article discusses the Dietary Guidelines for Americans (DGA) in support of a total diet approach to achieving diet and health goals, especially as they relate to the obesity epidemic. However, some scientists and organizations have identified one food, food group, or nutrient as the cause of the obesity epidemic and recommend that simply reducing that food/food group/nutrient will solve the problem. This is simplistic and unlikely to be effective in long term management of the obesity problem. This article also acknowledges discrepancies in the literature and the lack of consensus opinions from systematic reviews. Failure to consider the evidence as a whole can lead to inaccurate reports which may, in turn, adversely influence clinical practice, public policy, and future research. This article also considers where the line should be drawn between individual choice and responsibility and public regulation. Using sugar sweetened beverages as an example, the article considers the lack of a consistent association between added sugars and weight in the literature and calls for policy recommendations that are based on science and emphasizes the need for evidence-based policies rather than policy-based evidence.

## 1. Introduction

Over the past three decades, the percentage of adults who are obese has doubled; the percentage of children who are overweight has doubled; and the percentage of adolescents who are overweight has tripled. More recently, however, there appears to have been a slowing of the rate of increase or even a leveling off, at least in some gender or racial/ethnic groups [1],[2]. Obesity rates in the United States (US) have reached unprecedented proportions. Today, 69% of adults, age 20 and older, are overweight or obese [3]. Some experts project that 75% of adults will be overweight with 41% obese by 2015 [4]. About one in three children are overweight or obese, nearly triple the rate in 1963 [3],[5]. Obesity in both children and adults is most prevalent among ethnic minority groups[6]–[9]. According to the Centers for Disease Control and Prevention, blacks had 51% higher and Hispanics, 21% higher obesity rates compared with whites [10]. The significant racial and ethnic disparities in obesity prevalence highlight the importance of implementing effective intervention strategies among the general US population.

Obese children tend to become obese adults [11]–[13]. Obesity contributes to the major causes of death in the US, including atherosclerotic cardiovascular disease, hypertension and stroke, type 2 diabetes, and some forms of cancer [14]–[16]. Obesity affects the quality of life, increases medical costs, and increases job absenteeism in adults [17]–[18]; direct and indirect costs associated with obesity in adults is estimated at $209 billion or 20.6% of US healthcare expenditures [18].

The obesity epidemic in the US and other industrialized countries has created the impetus to find an immediate and simple solution to a complex problem. The causes of obesity are rooted in environmental, cultural, and behavioral factors that make obesity easier to come by than it is to avoid or to treat. Yet, there are those who advocate simplistic approaches to curb the obesity epidemic—these include the idea that single foods, food groups, or nutrients are the single cause of obesity. Thus, they advocate that foods (or beverages), such as 100% fruit juice [19] or nutrients or food groups' rich in specific nutrients, such as carbohydrates [20] or fats [21], be eliminated from the diet. Many of these “solutions” to the obesity epidemic surface in the popular press [22],[23] and result in edicts from elected government officials to make immediate and groundless changes since [24] none of these solutions have a firm science-base. For example, consumption of 100% fruit juice has been denigrated in the popular press [25] and even by some health professionals [26] as contributing nothing but empty calories. However, a recent critical review showed that there was little evidence to link fruit juice consumption with weight in children [27]. Another simplistic solution to the obesity epidemic are the government directives for taxes on single foods or that regulate the amounts sold; these include the recently proposed national soda tax [28],[29] and New York City's attempts to limit the amount of soda sold in a single restaurant serving [24],[30]. Not only are these approaches not science-based, they are inconsistent with the broad based recommendations of the Dietary Guidelines for Americans (DGA) [31] which is the cornerstone of US federal nutrition policy and Food and Nutrition Service programs [32].

## 2. The Great Sugar Debate

Five leading scientists [33]–[37] presented data and debated scientific findings on weight at the “Fructose, Sucrose, and High Fructose Corn Syrup: Modern Scientific Findings and Health Implications” symposium, held April, 2012 at the Experimental Biology meetings in San Diego, California. These findings related to the metabolism and health effects of fructose, high fructose corn syrup, and sucrose. This symposium was held, in part, as a response to the growing controversy between investigators who have suggested that sugars, specifically sugar-sweetened beverages containing fructose, may be associated with serious health conditions, and those that believe this not to be the case.

Two speakers [33],[34] presented theoretical arguments with proposed metabolic pathways to support their claim that “sugar is toxic,” [25],[34] a “sweet poison that is making us fat” [38] or is “alcohol without the buzz.” [34]. The metabolic pathways presented were based on animal studies, isolated piece-meal findings, and in some cases, conjecture/speculation [25],[34]. These speakers argued that fructose exerts negative effects beyond its caloric contribution to the diet and that the metabolic effects of fructose are unique, that is different from sucrose and glucose.

Drs. Lustig and Bray proclaim that “a calorie is not a calorie” because some foods contain calories are metabolized in a way that produces negative health effects. Some of these health effects include: increasing the risk of heart disease, as well as contributing to obesity, type 2 diabetes, non-alcoholic fatty liver, hypertension, and gout [25]. These misleading claims have not been shown to have a solid evidence-base. The calorie-is-a-calorie idea dates to 1878 when Max Rubner established what he called the Isodynamic Law; in that law, the form of human energy intake is irrelevant to its effect on energy balance [41]. This belief was later applied to obesity by Carl Von Noorden's theory that common obesity was all about “calories-in-minus-calories-out.” [40] This theory has been well established and accepted since the 1900s but is now being challenged in the 21^st^ century [25].

The other speakers at this symposium [35],[39] clarified for the audience the well-established metabolic pathways related to the metabolism of sugars and provided detailed evidence on the metabolic equivalence of high fructose corn syrup and sucrose. Majority of the speakers concluded that the scientific evidence does not exist that uniquely links the metabolism of fructose from normally consumed sugars at typical doses to a variety of adverse health conditions. This conclusion is consistent with previous meta-analyses [41]–[43]. Moreover, research suggests a marked benefit from fructose for glycemic control when consumed in amounts normally found in fruit [44],[45]. Dr. Klurfeld ended the debate stating that “the claim that sugar is ‘toxic’ does not pass the test of face validity and that although Americans consume too much sugar, it is only one factor in a poor dietary pattern.” He also noted that regulating or taxing sugars is a “political decision and that insufficient nutritional data exists to justify such a decision” [37].

## 3. Dietary Guidelines for Americans: Total Diet Approach to Meeting Recommendations

The DGA provide science-based advice to promote health and reduce risk for major chronic diseases through diet and physical activity [46]. The 2010 DGA [31] replaced the earlier 2005 DGA [47], although the recommendations for the individual food groups were to all intents and purposes the same and both promoted a nutrient-dense diet. Nutrient dense foods were defined by the DGA as those foods and beverages that provide vitamins, minerals, and other substances that may have positive health effects with relatively few kilocalories (kcals) [31]. Energy imbalance resulting in overweight or obesity primarily results from excess energy intake and physical inactivity. Many Americans consume more energy than they need [46],[48], without meeting recommended intakes for specific nutrients. On average, adults and children fail to meet recommended intakes for calcium, potassium, fiber, and vitamin D; and the 2010 DGA has identified these nutrients as those of public health concern [46],[48]. Other nutrients, including magnesium, vitamin B12, folate, and iron were identified as “shortfall” nutrients or nutrients of concern for some sub-populations [46]. Americans also consume inadequate amounts of four nutrient-dense food groups—fruit, vegetables, whole grains, and low-fat or fat-free dairy products [46],[48]. 80% to 99% of Americans have usual intakes below the recommended servings of these four food groups [49].

The DGA, in common with other agencies, such as the Academy of Nutrition and Dietetics [50], the American Cancer Society [51], and the National Heart, Lung, and Blood Institute [52] support a total diet approach to achieving diet and health goals. The total diet approach is based on overall eating patterns that have important benefits and supply adequate nutrients within individual energy needs. The total diet approach [50],[53] promulgated by the DGA is a combination of foods and beverages that constitutes an individual's complex dietary intake, on average, over time and provides appropriate intakes of energy and nutrients for that individual. This approach is intended to help Americans personalize dietary recommendations and to offer flexibility based on individual and cultural preferences [53]. Consumption patterns that meet energy needs can help individuals reach and maintain a healthy weight; patterns that help individuals meet nutrient needs are also associated with reduced risk of chronic diseases [31]. Foods are not consumed in isolation but in combination; thus, their effects on weight and other health parameters are interrelated and cumulative. Thus, any recommendation that singles out one food, food group, or nutrient as the single constituent to solve the obesity problem is naive and unlikely to be effective in the long term.

The total diet approach [50] does not label foods as “good foods or bad foods” because this could cause many people to abandon efforts to make dietary improvements. Eighty-two percent of US adults reported not wanting to give up on foods they like as a reason for not eating healthier [50]. Thus, “focusing on variety, moderation, and proportionality in the context of a healthy lifestyle, rather than targeting specific nutrients or foods, can help reduce consumer confusion and prevent unwarranted reliance on dietary supplements” [50].

## 4. Targeting Single Foods or Nutrients as a Solution to the Obesity Epidemic

Added sugars and sugar-sweetened beverages (SSB) are one nutrient/food group that has been vilified as a major cause of the obesity epidemic [33],[54]. Similar to the Australian Paradox [55], there has been a substantial decline in intakes of added sugars and SSB from 1999–2008 in the US which is the same time frame that overweight and obesity have increased [56]. In contrast, there was a significant increase in low-calorie sweeteners from 1999–2008 in the US [57]. Among children and adolescents 2 to 19 years of age, average energy intakes decreased over a 12-year period (1999–2000 through 2009–2010) [58]. The average intake for boys decreased by 7% and for girls 4%. With this decrease in energy intake a shift in macronutrient intakes was observed. The percent energy from protein increased, percent energy from carbohydrates decreased, and, percent energy from total fat changed very little. A similar analysis was conducted with adults 20–74 years of age from 1971–1975 to 2009–2010 [59]. After decades of increases (1971–2003), energy intake in adults decreased significantly between 2003 and 2010. It remains unclear if the changes in the prevalence of obesity were associated with changes in macronutrient intakes in children and adolescents during these time frames. Yet, it is noteworthy that reported energy intakes have decreased over a time period when the prevalence of obesity was steadily increasing.

Between 2006–2012, eighteen critical reviews or formal meta-analyses looking at the relationship between added sugars, especially SSB, and obesity have been published. Nine review studies concluded that there was strong evidence that SSB were positively associated with the weight status [46],[60]–[67]. However, the other nine reviews [68]–[76] concluded that the evidence was inconclusive. One systematic review was conducted to examine the association between the prevalence of overweight and obesity in more than 137,000 youths from 34 countries and their relationships with physical activity and dietary patterns [77]. The authors concluded that overweight status was not associated with the intake of fruits, vegetables, or soft drinks.

The findings from well-conducted systematic reviews and meta-analyses should all lead to the same conclusion; however, this is clearly not the case. Failure to consider the evidence as a whole, can lead to inaccurate statements which may inappropriately influence clinical practice, public policy, and future research.

There are several reasons for discrepancies in the published findings. As quoted by Gibson [70] these include:

- Differing definitions of SSB
- Differing units for serving size and frequency of consumption
- Unreliable dietary assessment methods
- Narrow focus on SSB with inadequate assessment of other diet components, nutrients or energy
- Measurement error due to response bias, for example with self-reported weight and height
- Poor or no measurement of physical activity
- Inadequate exploration of confounders or moderating factors in the analysis (for example, baseline Body Mass Index [BMI], ethnicity, baseline diet, misreporting)
- The high degree of inter-correlation (multi-collinearity) among some dietary factors
- Inconsistent evidence between subgroups
- Underpowered studies, where no conclusions can be drawn
- Possibility of publication bias towards positive studies

Mattes et al. [71] conducted a systematic review and meta-analysis of six randomized experiments that attempted to reduce consumption of SSB in an effort to reduce BMI. The authors concluded that the current evidence from randomized controlled trials did not demonstrate conclusively that reducing SSB reduces BMI in the general population. The authors recommended adequately powered randomized controlled trials with an overweight population.

Weed et al. [78] conducted a systematic review of the methodological quality of published reviews of analytic epidemiologic studies that examined consumption of SSB. Less than one-half of the reviews they examined documented which studies were included and excluded, failed to critically assess the quality of those studies, or provided conclusions based on clearly described methods. A well-conducted systematic review provides readers with a precise summary based on a comprehensivesynthesis of the available evidence and this is missing in many of the reviews of SSB.

Another reason why there are discrepancies in the literature related to consumption of added sugars and obesity may be due to “citation bias.” Cope and Allison [79] looked at secondary reporting of original research looking at two of the six randomized experiments that attempted to reduce consumption of SSB in an intervention; studies by James et al. [80], and Ebbeling et al. [81]. One hundred and ninety-five other papers have cited the James study and 45 papers have cited the Ebbeling study. Both reviews showed no significant change in adiposity or BMI as a result of the intervention. According to the authors, the majority of the studies (83.3% for James and 66.7% for Ebbeling), described results in a deceptively positive manner; that is, reduction of SSB showed beneficial effects on obesity outcomes. Some were obviously factually incorrect; that is, they described the results as showing an effect on obesity outcomes when no statistically significant effects were observed. A result of “citation bias” or statements that inaccurately describes results of studies may unfortunately influence clinical practice, public policy, or future research. This has been the case with SSB.

**Figure 1.**
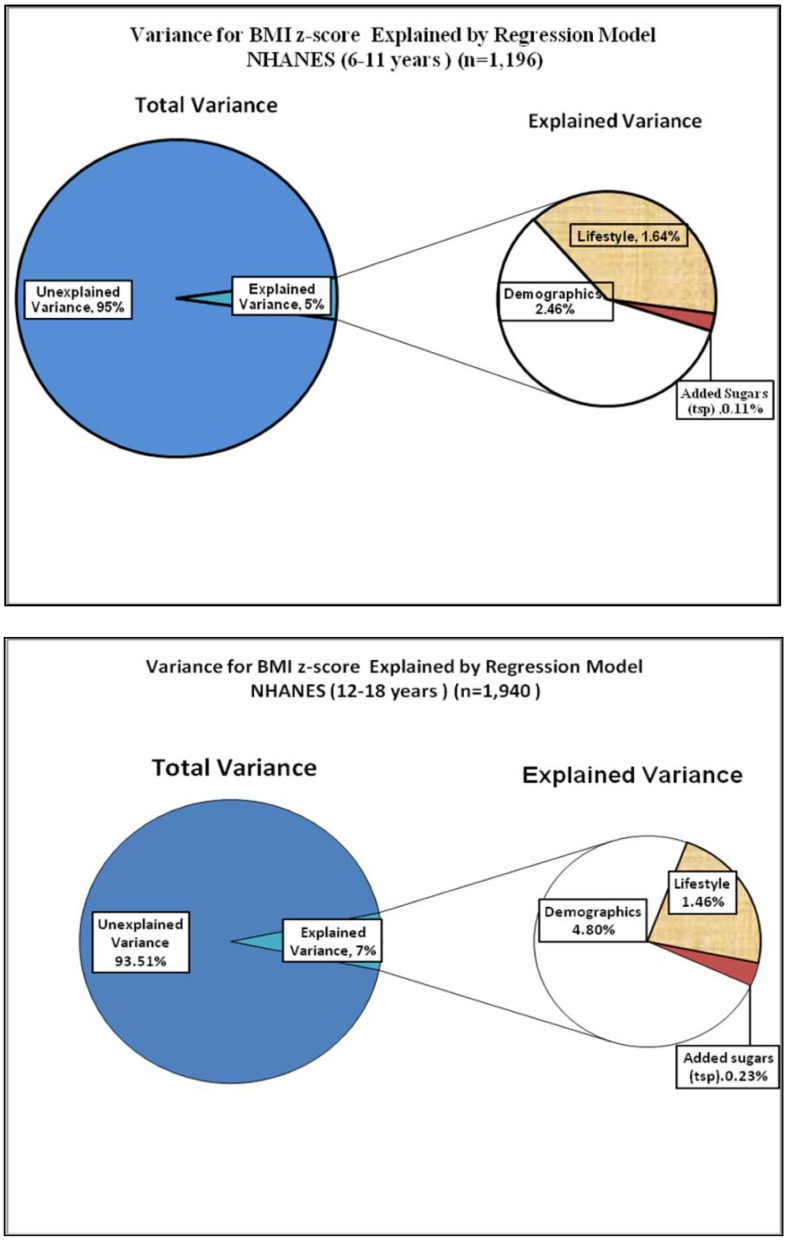
Variance for BMI z-score Explained by Regression Model NHANES 6–11 years old and 12–18 years old.

The majority of the studies showing a positive association between intakes of added sugars and overweight have neglected to report what percent of the variance was explained in this association. However, one study [82] examined the relationship between added sugars and weight status on a nationally representative sample of children, 6 to 18 years of age (*n* = 3136). Key findings from this study were: 1) the mean daily intake of added sugars was 23 tsp., representing 16.8% of total energy consumed; 2) the mean intake of added sugars was not associated with weight status; 3) normal weight children and adolescents had the highest intakes of added sugars; and, 4) consumption of added sugars explained virtually none of the variance in BMI z-scores for children (0.11%) and adolescents (0.23%) (Figure 1). The small amount of variance in BMI explained by diet has been confirmed by another study on children [83]. The lack of association between added sugars and weight was confirmed in another US national study [84].

Another major confounder in understanding the association of single foods with weight status is the issue of multi-collinearity of foods in the diet. Given that a variety of foods are consumed in the diet, singling out a single food can result in misleading results if the other foods are not taken into consideration. Individuals who drink SSB may do so at the expense of decreasing consumption of milk [85],[86]. It has also been shown that decreased milk consumption is associated with increased weight [87],[88]. Hugh Pedoe has been quoted as saying “If you eat more of one thing, you eat a lot less of something else. So, for every theory saying this disease is caused by an excess in x, you can produce an alternative theory saying it's a deficiency in y.” [89] To translate this into practical terms, if SSB are consumed, it's likely that less milk is consumed. So, for every theory saying that obesity is caused by an excess in consumption of SSB, one can produce an alternative theory saying it's a deficiency in drinking milk or another beverage. The issue of adjusting for other foods and beverages correlated with a single food has not been addressed by the majority of the studies looking at the association between consumption of SSB and weight. Thus, what appears to be a direct association may not be true because the association may be from another food or a combination of foods typically consumed in the diet. Finally, the overall assumption being made is that the association between SSB and weight is universal and “one-size fits all.” Yet, studies show that eating patterns vary by age, ethnicity, gender, and socioeconomic status [90],[91]. Although most studies control for these demographic differences in the analyses, few studies control for other foods consumed in a diet that are highly correlated with the dependent variable being studied. These findings beg the question: Should we be making policy recommendations based on intuition versus science, inconsistent findings, lack of consensus from systematic reviews, and, when the amount of variance explained in weight status is less than 1% for a single food or nutrient?

## 5. Government Interventions: A Soda Tax to Regulating Amounts of Soda Sold

In an attempt to partially mitigate the obesity epidemic, a soda tax has been proposed [92]–[94]; however, there is no conclusive evidence that such a tax will actually decrease obesity [95]. Any impact would depend, in part, on whether individuals would continue to buy the product or replace soda with other foods with an equivalent or even higher number of kilocalories [95]. An important take-away message from the current literature is that consumer's substitution behavior is very important in understanding the effects of food and beverage taxation [96]. The available evidence is that substituting other high energy drinks or foods would blunt the effectiveness of soft drink taxes in reducing obesity [28],[96].

A number of published studies by economists have looked at the impact of a soda tax on weight—but once again there is no consistent evidence that a soda tax will significantly reduce obesity [96],[97]. Some authors concluded that even large SSB taxes would have little impact on weight outcomes based on findings that reductions in energy intake from SSB would be largely offset by increased energy intake from other beverages [28],[29].

So, what could be an “unintended consequence” with a soda tax? A study conducted by the economists, Dharmasena and Capps [28] found that with a soda tax, there was a decrease in consumption of SSB but with a trade off with an increase in consumption of fruit juice. The unintended consequence was an increase in energy from consumption of fruit juice. A study by Fletcher et al. [29] reported an increase in energy from high-fat milk. The effects of a soda tax on health may depend primarily on the substitution patterns of those who stop buying soda as a result of the tax [28],[96]. Existing literature is uncertain about the effects of a soda tax on other beverage or food consumption, in part because no direct studies are available. Milk and fruit juice are nutritious beverages. However, the increase in milk or fruit juice consumption in response to soda sales tax could offset the benefits of a reduction on sugary soda taxes on weight because of the similar energy content of soda and milk or juice. Further research is needed to examine the actual impact of levels and structure of taxation on weight as well as any beverage substitution patterns that could offset benefits from taxation.

The question remains whether efforts to restrict the availability of SSB serves as a useful complement to ongoing policies aimed at reducing obesity. The question of school vending machine-available foods is an example; although their contents have been vilified as a contributor to childhood obesity, children actually drink more SSB at home than they do at school [98]. It's clear that most children have multiple venues to purchase SSB and other potential beverage substitutes outside the lack of availability in school vending machines.

**Figure 2.**
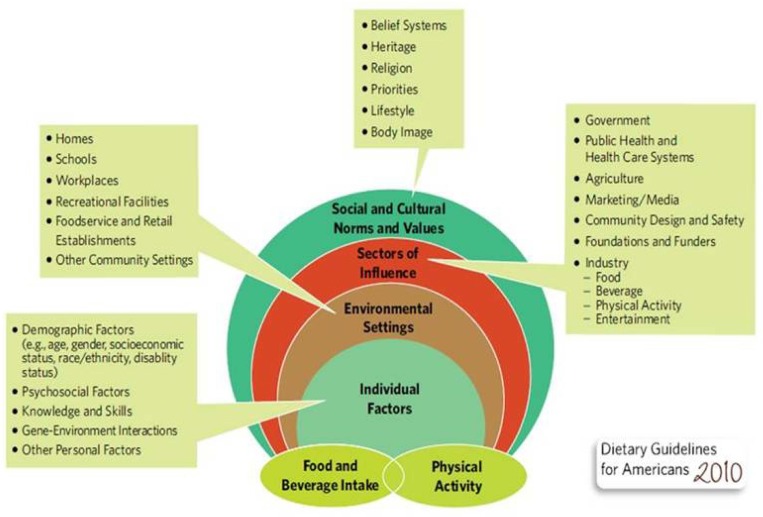
US Department of Agriculture, US Department of Health and Human Services. Dietary Guidelines for Americans, 2010. http://www.cnpp.usda.gov/Publications/DietaryGuidelines/2010/PolicyDoc/Policy Doc.pdf. (Accessed on May 1, 2013).

A major question is: Is public-paternalism an appropriate way to manipulate diet? If so, why don't we tax doughnuts, cookies, candy, or chips or other fried foods? A study conducted by Cohen, et al .[99] reported that the mean number of kcals from salty snack/cookies/candy combined ranged from 271 to 413 kcals compared to 74 to 199 kcals coming from soda. In the 2010 DGA report [46], the major food source of energy were grain based desserts (*e.g.* cakes, cookies, donuts, pies). In another study by Reedy et al. [100] the top sources for energy for children 2–18 years of age were grain desserts (138 kcal/day), pizza (136 kcal), and soda (118 kcal). So why don't we tax all these foods? Where should the line be drawn between individual choice and responsibility and public regulation? Should we support a society that creates forced food and beverage choices and limits personal freedom? This amplifies the need for evidence-based policies rather than policy-based evidence.

The foundation for a good health policy should not be a soda tax, which can lead to the good food and bad food approach for treating symptoms and not causes. The causes of overweight and obesity are multi-factorial, rooted in environmental, behavioral, and cultural factors that make obesity easier to come by than to avoid. The Social Ecological Model [46] demonstrates the complexities of understanding what influences the energy equation-both a balance of energy intake and energy expenditure (Figure 2). This model involves individual factors, environmental settings, sectors of influence, and social and cultural norms and values. A detailed discussion of these factors have been recently reviewed in a Position paper of the Academy of Nutrition and Dietetics on a Total Diet Approach to Healthy Eating [50].
